# Pharmacogenetic-Based Efavirenz Dose Modification: Suggestions for an African Population and the Different CYP2B6 Genotypes

**DOI:** 10.1371/journal.pone.0086919

**Published:** 2014-01-31

**Authors:** Jackson K. Mukonzo, Joel S. Owen, Jasper Ogwal-Okeng, Ronald B. Kuteesa, Sarah Nanzigu, Nelson Sewankambo, Lehana Thabane, Lars L. Gustafsson, Colin Ross, Eleni Aklillu

**Affiliations:** 1 Department of Pharmacology and Therapeutics, College of Health Sciences, Makerere University, Kampala, Uganda; 2 CIHR, Canadian HIV Trials Network, Vancouver, British Columbia, Canada; 3 School of Pharmacy, Union University, Jackson, Tennessee, United States of America; 4 Departments of Clinical Epidemiology and Biostatistics, Pediatrics and Anaesthesia, McMaster University, Hamilton, Ontario, Canada; 5 Division of Clinical Pharmacology, Department of Laboratory Medicine, Karolinska University Hospital-Huddinge, Karolinska Institutet, Stockholm, Sweden; 6 Division of Translational Therapeutics, University of British Columbia, Vancouver, British Columbia, Canada; 7 Biostatistics Unit, St. Joseph’s Healthcare—Hamilton, Hamilton, Ontario, Canada; Hospital Italiano de Buenos Aires, Argentina

## Abstract

**Background:**

Pharmacogenetics contributes to inter-individual variability in pharmacokinetics (PK) of efavirenz (EFV), leading to variations in both efficacy and toxicity. The purpose of this study was to assess the effect of genetic factors on EFV pharmacokinetics, treatment outcomes and genotype based EFV dose recommendations for adult HIV-1 infected Ugandans.

**Methods:**

In total, 556 steady-state plasma EFV concentrations from 99 HIV infected patients (64 female) treated with EFV/lamivudine/zidovidine were analyzed. Patient genotypes for CYP2B6 (*6 & *11), CYP3A5 (*3,*6 & *7) and ABCB1 c.4046A>G, baseline biochemistries and CD4 and viral load change from baseline were determined. A one-compartment population PK model with first-order absorption (NONMEM) was used to estimate genotype effects on EFV pharmacokinetics. PK simulations were performed based upon population genotype frequencies. Predicted AUCs were compared between the product label and simulations for doses of 300 mg, 450 mg, and 600 mg.

**Results:**

EFV apparent clearance (CL/F) was 2.2 and 1.74 fold higher in CYP2B6*6 (*1/*1) and CYP2B6*6 (*1/*6) compared CYP2B6*6 (*6/*6) carriers, while a 22% increase in F1 was observed for carriers of ABCB1 c.4046A>G variant allele. Higher mean AUC was attained in CYP2B6 *6/*6 genotypes compared to CYP2B6 *1/*1 (p<0.0001). Simulation based AUCs for 600 mg doses were 1.25 and 2.10 times the product label mean AUC for the Ugandan population in general and CYP2B6*6/*6 genotypes respectively. Simulated exposures for EFV daily doses of 300 mg and 450 mg are comparable to the product label. Viral load fell precipitously on treatment, with only six patients having HIV RNA >40 copies/mL after 84 days of treatment. No trend with exposure was noted for these six patients.

**Conclusion:**

Results of this study suggest that daily doses of 450 mg and 300 mg might meet the EFV treatment needs of HIV-1 infected Ugandans in general and individuals homozygous for CYP2B6*6 mutation, respectively.

## Introduction

Efavirenz (EFV) is currently the most widely used non-nucleoside reverse transcriptase inhibitor (NNRTI) for HIV patients, particularly during co-treatment with rifampicin [1]. As a result EFV has been extensively used as part of highly active antiretroviral therapy (HAART). Despite extensive clinical experience with EFV, unpredictable inter-individual variability in efficacy and toxicity remain important limitations associated with its use. EFV exhibits significant inter-individual pharmacokinetic variability as well as a narrow therapeutic window, with plasma concentrations >4 µg/mL being associated with more central nervous system (CNS) toxicity while the rate of virologic failure increases with concentrations <1 µg/mL [2]. Consequently, EFV therapeutic drug monitoring has been recommended [3]. Therapeutic drug monitoring is however not universally achievable, as it is not feasible in resource constrained settings. Among the factors affecting EFV pharmacokinetics are ethnicity, host genetic factors, gender, body weight, drug interactions, binding to plasma proteins, hepatic impairment, disease status and pregnancy [4]–[7].

EFV undergoes oxidative hydroxylation primarily by CYP2B6 to 8-hydroxyEFV as a major metabolite, and to 7-hydroxyEFV as a minor metabolite [8]. CYP2B6 516G>T (*6) has particularly been reported to be associated with a pronounced reduction in enzyme activity and elevated EFV plasma concentrations in studies conducted on different populations [6], [9]–[11]. There is evidence that CYP2B6*6 variants are poor metabolizers and therefore at risk of high EFV plasma concentrations and related consequences such as adverse drug reactions often leading to poor compliance. The EFV alternative metabolic pathways: CYP2A6, CYP3A4/A5 and UGT2B7 appear to influence EFV elimination independent of CYP2B6. Additionally, CYP2B6 c.136A→G) and ABCB1 c.4036 A→G influence both EFV plasma and intracellular concentrations [6], [12].

CYP2B6*6, CYP2B6 (c.136A→G) and ABCB1 c.4036 A→G are expressed differently by various populations. In our previous study we found *CYP2B6 516G>T* and 785A>G were in complete linkage disequilibrium in Ugandans with an overall expression of the variant allele CYP2B6*6 in at least 50% of the population [6], compared to 3.4% in the western/Caucasian population. The SNP frequencies for CYP2B6 c.136A→G and ABCB1 c.4036 A→G in the same population were 13.6% and 15.8% respectively [6]. Most Sub-Saharan African populations are either heterozygous or homozygous for defective variant alleles of CYP2B6 *6 [6], [13] that might result in different features of EFV kinetics and clinical response than other races. Consequently EFV population based dose stratification may be beneficial. However, dose modification needs to be based upon well-derived exposure measures that take into account clinically relevant genetic factors.

A pharmacokinetic-pharmacogenetic model was constructed using steady state EFV concentrations in HIV-1 infected patients to: 1) describe genetic effects on EFV steady state pharmacokinetics, 2) estimate the population pharmacokinetic parameters for EFV exposure, and 3) simulated optimal EFV doses for HIV-1 infected Ugandans and CYP2B6*6 and ABCB1 c.4046A>G variants so as to guide dose selection during treatment of these populations.

## Subjects and Methods

The current study was conducted in accordance with the Declaration of Helsinki. Ethical approval was obtained from The Uganda National Council for Science and Technology. Written informed consent was obtained from all participants. The dataset contained 556 EFV concentration values collected from 99 HIV/AIDS patients (64 females) over 252 days from the 14^th^ day of initiation of EFV based HAART. All subjects received oral daily dose of 600 mg EFV (Stocrin®; Merck, Sharpe & Dohme, Whitehouse Station, NJ, USA) plus zidovidine/lamivudine (150 mg/300 mg). In addition, subjects received prophylactic trimethoprim/sulfamethoxazole treatment. Mid-dose EFV plasma concentrations samples (11–18 hours after the last dose) were collected on about five different occasions per subject over the study period. CD4 counts and HIV-1 RNA cells/ml measures were performed at baseline, months 3 and 6. Participant genotypes for CYP2B6 (*6 & *11), CYP3A5 (*3, *6 & *7) and *ABCB1* (*c.4046A>G* and *c.3435C>T*) were also determined.

## Bio-Analysis

### Efavirenz Pharmacokinetic Analysis

Blood samples were collected into EDTA tubes and prepared for analysis by centrifugation at 3000 rpm for 10 min and stored at −70°C until HPLC analysis was performed. Plasma EFV was determined by reverse phase HPLC with UV-detection as described (12). The HPLC instrument, Agilent series 1100, consisted of a column compartment G1316A, Degasser G132A, Quat pump G1311A, and an auto-sampler ALS, G1329A, and G1315B diode array detector. The column used was Ace3C18, 3 µm 50×30 mm (Advanced Chromatography Technologies, Aberdeen, Scotland). The standard used was EFV (99.9%), supplied by the WHO Collaborating center for chemical reference substances through Apoteket AB Stockholm, Sweden. The retention time for EFV was 2.42 minutes as detected at UV-VIS 1, 210 nm, UV-VIS 2, 220 nm. This method was linear, with a within-day coefficient of variation of 3.2, 3.3 and 5.1% at concentrations of 2.0 mM (*n = *17), 8.0 mM (*n = *17), and 20 mM (*n = *16), respectively, and a between-day coefficient of variation of 4.1% (*n = *50).

### Genotyping

Genomic DNA was isolated from peripheral blood leukocytes using QIAamp DNA Maxi Kit (QIAGEN GmbH. Hilden. Germany). All participants were genotyped for CYP2B6*6 and *11, CYP3A5*3,*6 and *7 and *ABCB1* (3435CT and rs3842). SNP selections, apart from ABCB1 (3435C>T), was based in their role in EFV pharmacokinetics according to our previous report (12). *ABCB1* 3435C>T was selected on basis of previous conflicting reports on its role in pharmacokinetics and pharmacodynamics of ART (24–28). Allelic discrimination reactions were performed using TaqMan® (Applied Biosystems, CA, USA) genotyping assays: (C___7586657_20 for *ABCB1* 3435C>T, C___7817765_60, for *ABCB1* rs3842T>C, C__29560333_20, for *CYP2B6* 516G>T [CYP2B6*6 ], for *CYP2B6* 136A>G [CYP2B6*11], C__26201809_30 for *CYP3A5* 6986A>G [CYP3A5*3], C__30203950_10 for *CYP3A5* 14690G>A [CYP3A5*6]) and C__32287188_10 for *CYP3A5* g.27131_27132insT [CYP3A5*7] on ABI 7500 FAST (Applied Biosystems, Foster City, CA). The final volume for each reaction was 10 µl, consisting of 2x TaqMan Universal PCR Master Mix (Applied Biosystems), 20 X drug metabolising genotype assay mix and 10 ng genomic DNA. The PCR profile consisted of an initial step at 50°C for 2 min and 50 cycles with 95°C for 10 minutes and 92°C for 15 sec.

## Data Analysis

### Pharmacokinetic Model Development

A population PK model of EFV was built using nonlinear mixed-effect modeling (NONMEM) (version 7.2.0). The software ggplot2 (Version 9.3.1), PsN 3.4.2, and SAS 9.2 were used for dataset construction, graphical, and statistical analysis. The first-order conditional estimation method (FOCE) was used. A one-compartment model with first-order absorption and elimination (specified to NONMEM by the ADVAN2 and TRANS2 routines) was assumed. Since the data lacked observations within the absorption phase, the absorption rate constant (*Ka*) could not be estimated but was instead fixed at 0.3 h^−1^, a value previously reported [14], [15]. Estimated fixed-effect PK parameters included the apparent clearance (CL/F), relative bioavailability (F1) between ABCB1 groups, and the apparent distribution volume (V/F). Model discrimination was based on relative objective function values (OFV), precision of parameter estimates, and goodness-of-fit plots. Interindividual variability (IIV) was included on Cl/F and V/F with exponential error models. Residual error was described with an additive plus proportional error model.

### Covariate Analysis

Covariate analysis was performed using a forward-selection (α = 0.05) followed by backward elimination (α = 0.01) method. Albumin, gender and pharmacogenetic covariates including *CYP2B6* (*6 and *11) and *ABCB1* (*c.4046A>G)* were tested in the model. Each covariate-parameter relationship was first tested in a univariate manner. Covariates with two and three degrees of freedom were included in the forward selection if they reduced the OFV by at least 5.99 and 7.81 respectively, corresponding to a p-value of <0.05 for a χ^2^ distribution. The full covariate model was reached when the addition of further covariate-parameter relationships did not decrease the OFV to the specified criteria. The covariate-parameter relationships were re-examined in the backward deletion step in a manner similar to the forward inclusion step but reversed and with a more conservative significance level of α = 0.01. In addition to significantly reducing the OFV, the standard error on the covariate prediction had to be ≤30% of the predicted value.

### Estimates of Exposures

For each patient, EFV area under the curve was derived from the estimated individual pharmacokinetic parameter estimates as shown in **Equation 1**.
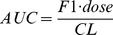
(1)


Typical group values of F1 and empiric Bayesian estimates of clearance were used in the computation of AUC. The doses needed to achieve comparable exposure in the different population subgroups were calculated using **Equation 2**.
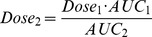
(2)


### Pharmacokinetic Simulations

PK simulations were performed for 500 datasets of 99 patients each, with the same genotype covariate frequencies as the original dataset. Fixed and random model effects were set equal to the final model. Doses of 300, 450, and 600 mg were simulated for each of the six possible CYP2B6*6 and ABCB1 (c.4046A>G) combinations and their frequencies in the study population. AUC was calculated for each simulated individual and summary statistics are presented.

### PK/PD Associations

Efficacy was measured in terms of immunological recovery (change between baseline and last measured CD4 counts or CD4 counts on days 84, 168 and >200) and virologic decay to below detection or <40 copies per milliliter by day 84. Correlations between AUC and change in efficacy were explored graphically.

## Results

Overall the pharmacokinetic dataset contained 556 EFV concentration values collected from 99 HIV/AIDS patients (n = 64 females) over days 252 of daily treatment with EFV based HAART. Mean (SD) bodyweight and age were 55.1(8.0) kg, 37.4 (7.6) years, respectively. The baseline mean (SD) serum albumin, alanine aminotransferase, urea and estimated creatinine clearance were 3.87 (0.79) g/dL, 17.79 (10.18) u/L, 2.85 (1.27) mMol/L and 78.9 (24.27) µmMol/L respectively. Other baseline characteristics and dose relevant genotype information on study subjects are summarized in **Table 1**. The population allelic frequencies of SNPS without implications for EFV dose modification that included; CYP2B6 *11, CYP3A5 (*3, *6 & *7) and *ABCB1 c.3435C>T* did not differ from findings of our previous study [6].

**Table 1:** Baseline characteristics and CYP2B6*6 and *ABCB1* (*c.4046A>G)* genotypes of the study participants (n = 99).

Measures of disease status	
Log_10_ viral load (±SD)	4.95±0.71
CD4 cell count/mL(IQR)	147 (89–207)
**Genotype**	
CYP2B6*6 (rs 2279343, 3745274)	
*1/*1	31.3% (n = 31)
*1/*6	54.5% (n = 54)
*6/*6	14.1% (n = 14)
***ABCB1 -c.4046A>G(rs3842)***	
A/A	60.6% (n = 60)
A/G	34.3% (n = 34)
G/G	1% (n = 1)
Missing	4% (n = 4)

Missing was assigned to individuals whose genotypes were not determined. Data presented in the table are the assignments as analyzed.

**Figure 1:**
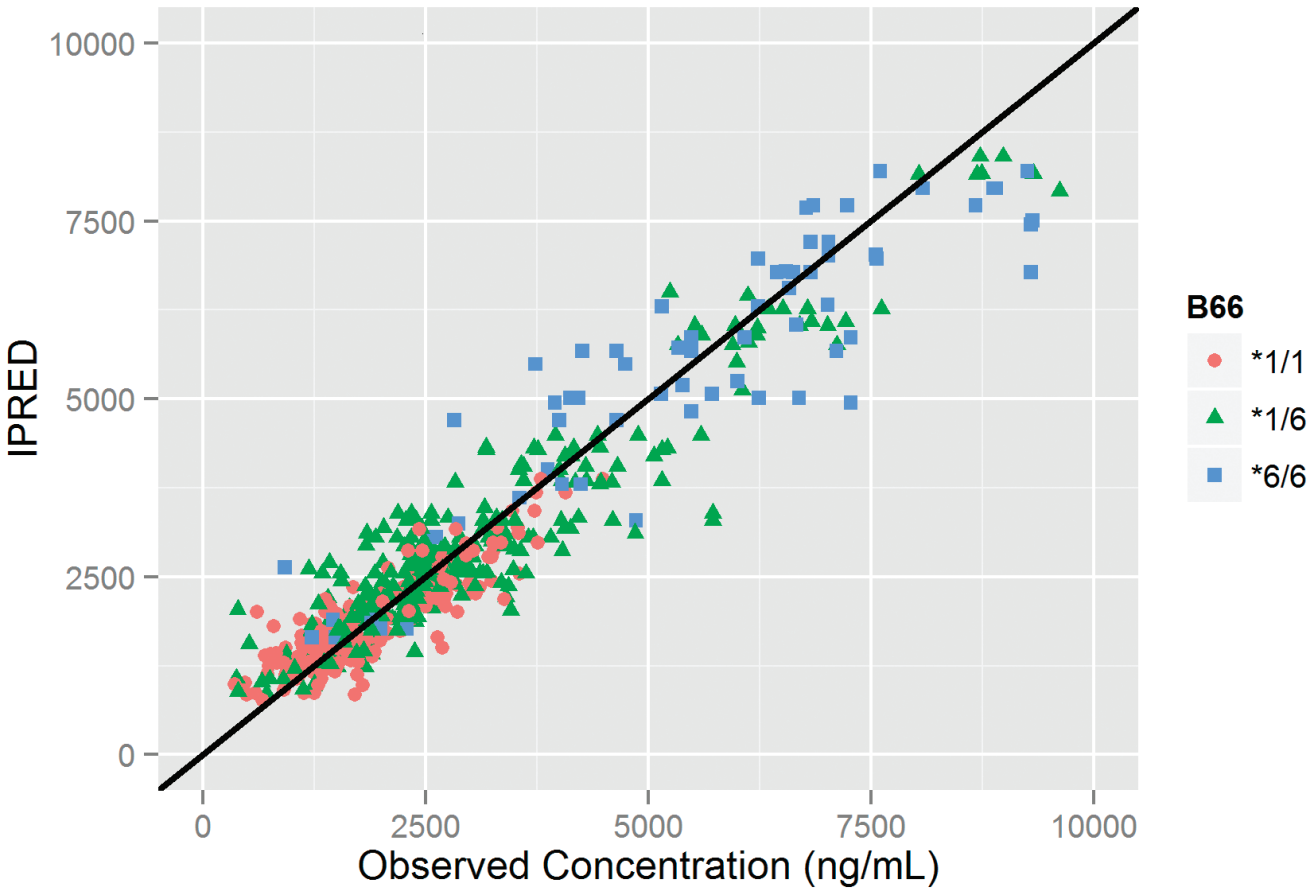
Goodness of Fit. Individual predicted EFV concentrations (IPRED) versus observed concentrations, by CYP2B6*6 genotype (B66).

**Figure 2:**
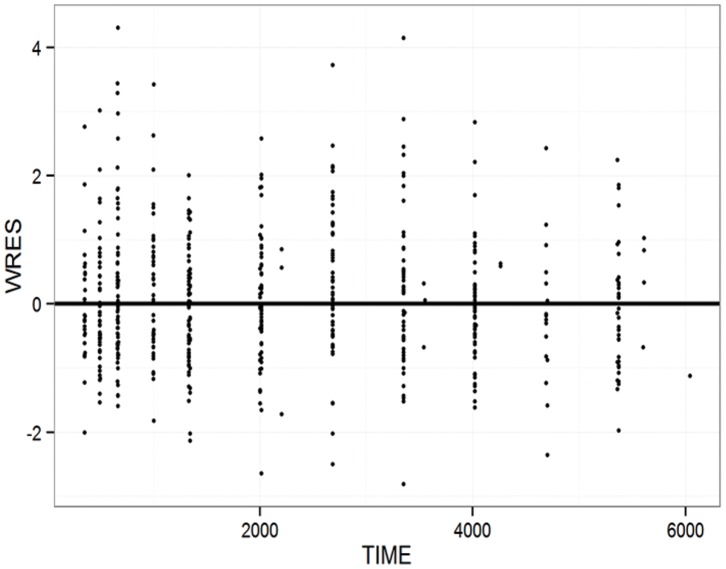
The individually weighted residuals (WRES) are plotted *vs.* time. The dashed line is the zero reference line while the solid line is a smooth nonparametric regression line. The plot demonstrates a good fit of all time point concentration data by the model.

A one-compartment model with first-order absorption described our data well, as can be seen **Figure 1** and **Figure 2**. All of the highest concentration values, those between 5,000 and 10,000 ng/mL, arose in patients expressing the variant CYP2B6*6 allele. The effects and statistical importance of covariates identified in the study population on pharmacokinetic parameter estimates are depicted in **Table 2**. The final model pharmacokinetic parameters are reported in **Table 3**. Notably, EFV post-induction CL/F was 2.2 and 1.7 fold higher in CYP2B6*6 (*1/*1) and CYP2B6*6 (*1/*6) compared CYP2B6*6 (*6/*6) carriers, while a 22% increase in F1 was observed for ABCB1 c.4046A>G variants.

**Table 2:** Summary of significant factors in the covariate analysis; forward inclusion (α = 0.05) followed by backward elimination (α = 0.01).

Covariate-Parameter Relationship (functional form)	ΔOFV	p-value
**Forward Inclusion**		
CYP2B6*6– CL (shift for heterozygous and wild-type)	30.359	2.34×10^−7^
*ABCB1* (*c.4046A>G* – F1 (shift)	15.883	0.0012
**Backward Elimination**		
*ABCB1* (*c.4046A>G* – F1 (shift) CYP2B6*6– CL (shift for heterozygous and wild-type)	13.295 25.431	0.004 3.004×10^−6^

**Table 3:** Final model pharmacokinetic parameters.

Final Pharmacokinetic Model	Parameter	SE(%CV)
Ka (hr^−1^)	0.300 Fixed	N/A
V (L)	94.5	24.4%
CL (L/hr) – CYP2B6*6 (*6/*6) – Homozygous Mutant	4.54	11.9%
CL (L/hr) – CYP2B6*6 (*1/*6) – Heterozygous Mutant	7.92	33.5%
CL (L/hr) – CYP2B6*6 (*1/*1) – Wild-Type	9.99	26.8%
F1 - ABRS 1&2	1 Fixed	N/A
F1 - ABRS 3	0.780	40.7%
F1 - ABRS 4	0.513	30.4%
IIV CL	0.134	19.9%
RV – Proportional (%CV)	17.3	26.3%
RV – Additive (ng/mL)	348	31.3%

Ka = Mean population absorption rate constant, V = Mean population Volume of distribution, CL = Mean population clearance, F1 = Bioavailability fraction, IIV CL = inter-individual variability on Clearance in the population, RV = residual variability.

**Figure 3:**
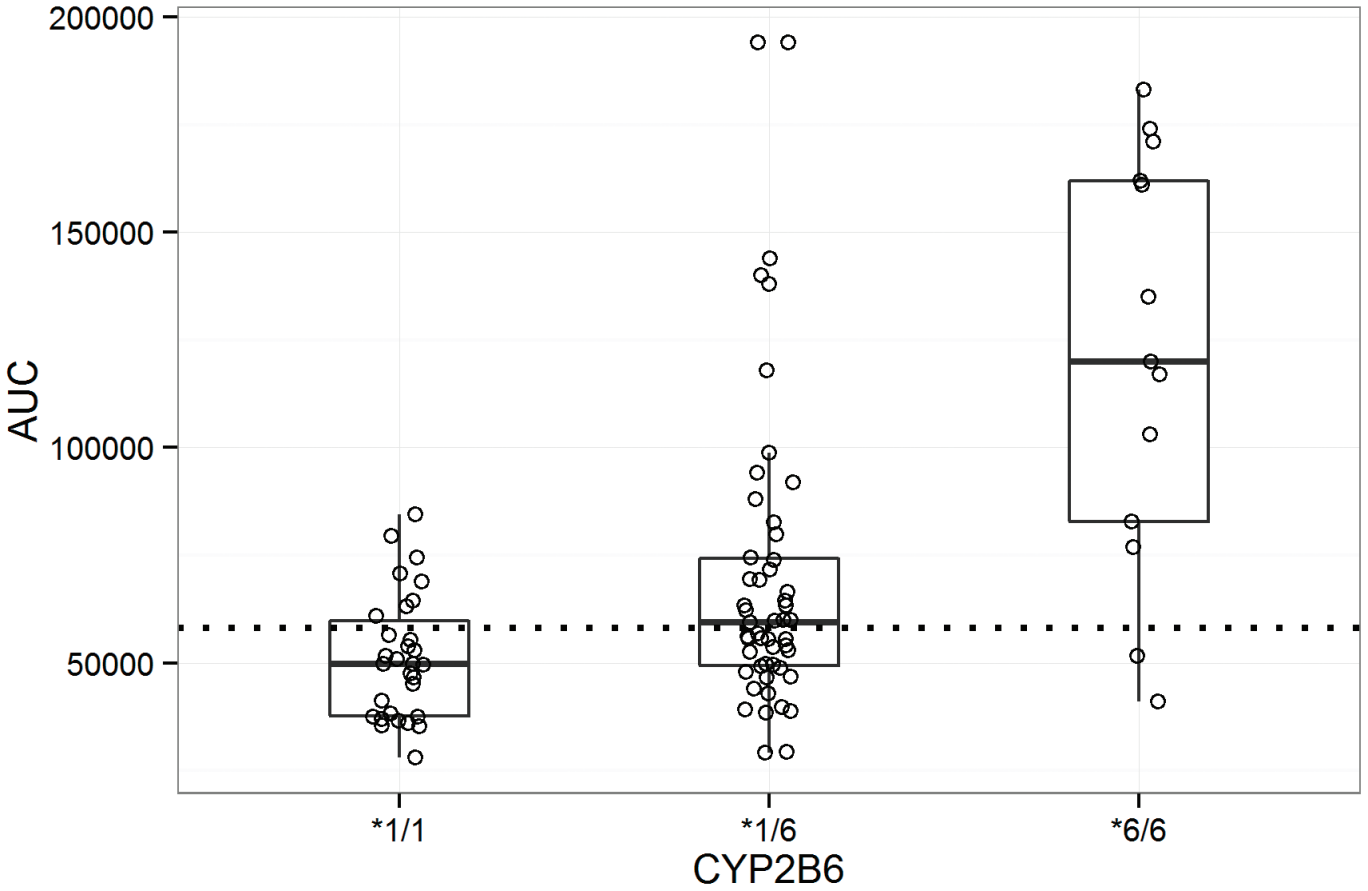
Distribution of estimated patient AUC values by CYP2B6 genotype. CYP2B6*1/*1, CYP2B6 *1/*6, and CYP2B6 *6/*6. Dotted line = the mean AUC value in the product label.

Estimated AUC values stratified by patient genotypes are presented in **Table 4** and plotted in **Figure 3**. The CYP2B6*6 genotype was found to be a major predicator of exposure to EFV with subjects homozygous for variant CYP2B6 *6 allele exhibiting a mean AUC of 12×10^4^ µg/L·h, more than 2 times mean AUC in the product label. Mean AUC values for both wild type and heterozygous mutants were within the exposure range observed during clinical studies reported in the product label. While plasma EFV exposure differed significantly between CYP2B6*6 (*1/*1)/homozygous wild type ABCB1 (*c.4046A>G)* and CYP2B6*6 (*6/*6)/mutant ABCB1 (*c.4046A>G)* (p<0.0001) no difference was observed between CYP2B6*6 (*6/*6)/mutant ABCB1 (*c.4046A>G)* and CYP2B6*6 (*6/*6)/homozygous wild type ABCB1 (*c.4046A>G)* p<0.53. Overall population simulation mean AUC was 72523 µg/L·h for the 600 mg dose group, 1.25 fold times the mean AUC in the product label of 58084 µg/L·h.

**Table 4:** Area under the curve (AUC) NONMEM estimate values for ABCB1 c. 4046A>G and or CYP2B6* 6 genotypes.

Genotype		n	Area Under the curve(µg/L·h) ×10^4^	
CYP2B6*6	*ABCB1*(*c.4046A>G)*		Mean (SD)	Range
*1/*6		54	6.999 (3.509)	2.917–19.380
*1/*1		31	5.160 (1.426)	2.796–8.456
*6/*6		14	12.221 (4.617)	4.115–18.334
*1/*6	**mut**	18	8.882(4.726)	3.876–19.378
	**wt**	35	6.147(2.216)	2.936–14.019
*1/*1	**mut**	8	5.658(1.582)	3.657–7.948
	**wt**	21	5.114(1.357)	2.796–8.455
*6/*6	**mut**	8	13.871(3.702)	7.677–18.334
	**wt**	4	11.070 (5.420)	5.155–17.367

The estimations are based on the predicted individual apparent clearance and bioavailability values.

mut = heterozygous plus homozygous variant, wt = homozygous variants.

Four patients did not have a reported ABCB1 genotype and one patient was ABCB1 G/G. These five subjects are not included in these summary statistics for ABCB1, but are included in the general CYP2B6*6 summary above.

By scaling exposures, EFV daily doses of 285 mg would be expected to achieve similar plasma exposure in individuals homozygous to CYP2B6*6 as reported in the EFV product label. Similarly, adjustments to a 487 mg daily dose of EFV would provide the typical Ugandan HIV-1 infected adult with exposure equal to the mean AUC in the drug label. Since these specific dose amounts are not achievable with existing market formulations, simulated exposures for EFV daily doses of 300 mg, 450 mg, and 600 mg are presented in **Table 5** to reflect similar doses that are achievable with the currently existing formulations.

**Table 5:** Simulation based AUC for 300

			Dose = 300 mg daily		Dose = 450 mg daily		Dose = 600 mg daily	
			Area Under the curve (µg/L·h) x10^4^					
		number of obs^a^	Mean (±SD)	95% CI	Mean (±SD)	95% CI	Mean (±SD)	95% CI
All participants		47000	3.63 (1.93)	1.57–7.58	5.44 (2.90)	2.35–11.06	7.25 (3.86)	3.14–14.75
ABCB1(c.4046A>G) wt	CYP2B6*6							
	*1/*1	17500	2.5 (0.94)	1.28–5.41	3.75 (1.41)	1.92–3.50	5.0 (1.88)	2.56–8.49
	*1/*6	2000	3.16 (1.17)	1.63–5.34	4.74 (1.75)	2.45–8.01	6.32 (2.34)	3.27–10 68
	*6/*6	10500	5.57 (2.14)	2.85–9.53	8.36 (3.21)	4.27–14.29	11.14 (4.28)	5.7–19.05
ABCB1(c.4046A>G) Mut								
	*1/*1	4000	3.20 (1.21)	1.65–5.41	4.80 (1.81)	2.48–8.11	6.41 (2.41)	3.31–10.81
	*1/*6	9000	4.05 (1.54)	2.10–7.05	6.07 (2.32)	3.14–10.58	8.10 (3. 09)	4.19–14.10
	*6/*6	4000	7.12 (2.72)	3.63–12.20	10.67 (4.09)	5.45–18.30	14.23 (5.45)	7.26–24.40

a The frequency of each group in these simulations reflects the proportional frequency of the groups in the observed patient dataset.

### Pharmacodynamic Evaluations

Baseline mean (SD) log_10_ HIV RNA copies per ml and CD4 counts were 4.972 (0.61) and 147.8 (81.0) respectively. Mean (SD) change from baseline CD4 counts at days 84, 168 and after 200 days of EFV based ART was 93.7 (87.2), 154.3 (83.0) and 206.1 (104.5) respectively, while CD4 change by last time of measurement was 177.9 (101.2). Six participants (6.1%) had HIV RNA >40 copies mL^−1^ after 84 days of ART. AUC values for these 6 patients, as well as their baseline and day 84 HIV RNA values are presented in **Table 6**.

**Table 6:** HIV viral loads at baseline and day 84 of treatment and AUC of study participants who failed to attain viral suppression to below detection by day 84.

AUC	HIV RNA >40 copies mL^−1^	
(µg/L·h)	Baseline	Day 84
27962	12170	6660
29359	356662	108
36573	534688	667
55512	900246	134
102750	123164	134
183340	18030	666

Data are arranged in order of increasing AUC.

Neither change in CD4 counts nor achievement of HIV RNA <40 copies mL^−1^ demonstrated a correlation with EFV exposure in this study. In part this may be because the pharmacodynamics response is so strong for most patients at all observed exposure levels on this combination HAART therapy.

## Discussion

Despite the existing evidence of extensive inter-ethnic EFV pharmacokinetic variability that is dependent on host-genetic-factors, its dosing in the sub-Saharan African region is to date based largely on data derived from studies conducted among Caucasians. We predicted EFV optimal dose for adult HIV-1 infected Ugandans based upon population genetic make-up and recommend a dose reduction from 600 mg to 450 mg daily dose. Findings of our study also reveal need for EFV dose reduction by 50% when treating patients homozygous for the variant CYP2B6*6 allele.

Consistent with existing reports on EFV pharmacogenetics, the current study demonstrates that variability in EFV pharmacokinetics is largely dependent upon CYP2B6*6 genotype [7], [9], [16]. Reduced EFV metabolism in individuals either homozygous or heterozygous for CYP2B6*6 ultimately results in increased plasma exposure to the drug with higher likelihood of EFV CNS related symptoms [16]. The black race has been associated with higher plasma EFV exposure [4], [17], consistent with our observation of the 1.25 fold higher AUC among HIV-infected Ugandans than the mean AUC in the product label. Higher frequency of EFV concentration dependent CNS adverse events that in turn influence treatment discontinuation rates [18] have previously been reported among Africans. Presumably the supra-therapeutic EFV exposure might play a role in adherence issues and consequent treatment outcomes of EFV based ART.

The current standard 600 mg/daily EFV dose is effective; but the higher EFV plasma exposure may pause a risk for toxicity and non-adherence particularly in CYP2B6 slow metabolizers without increased virologic and immunologic response. Recent prospective studies from African HIV patients indicated association of higher EFV plasma exposure with CNS adverse events [16], [19] and liver enzyme abnormalities [19], [20]. The recommended dose reduction to 450 mg daily according to this study that is plausibly explained by the higher allelic frequency of CYP2B6*6 in the study population also draw its justification from the higher frequency of EFV CNS symptoms among Africans. Our dose adjustment recommendation is also supported by a clinical case report of CYP2B6*6 heterozygous patient who successfully attained viral suppression and sustained it for more than 18 months on EFV dose of 400 mg daily as well as other studies including the recently concluded one by Puls et al [21]–[23]. Successful HIV viral suppression has been demonstrated at EFV doses of 400 mg and 200 mg daily among patients that exhibited supra-therapeutic plasma concentrations following 600 mg daily EFV dose.

The current study had a long follow up period of up to 252 days and pharmacokinetic data were collected at assumed steady state conditions. Six patients did not achieve viral suppression to below detection by day 84 of treatment. However, the lack of a correlation between their day 84 viral loads and either AUC or baseline viral load in the current study suggests other possible causes. Although there is need for studies designed to address this specific question, we postulate that some of the factors responsible for failure to achieve viral suppression to below detection in this particular study might include erratic adherence or intrinsic viral resistance. Considering that 4 (66.7%) of the individuals who failed to achieve viral suppression to below detection by day 84 had their AUC either within range or above the product label AUC, pre-treatment HIV viral resistance that was reported at a rate of 12.3% in Kampala Uganda [24] might have played a role.

Our findings may have extensive applications for most of the Sub-Saharan African region, and are supported by the high frequency of the defective CYP2B6*6 variant alleles among Africans as well as previous reports [21], [22], [25]. Among individuals homozygous for CYP2B6*6, simulated exposures for EFV daily doses of 300 mg (55712.3±21420.9 µg/L·h) were comparable to the product label (58084±23044 µg/L·h). This finding is supported by previous findings according to Mello et al and Gatanaga et al. who demonstrated sustained HIV viral suppression on an EFV dose of 200 mg daily [22], [26].

Even as sub-therapeutic concentrations of EFV are associated with treatment failure [2], [27], supra-therapeutic EFV plasma concentrations may lead to poor adherence, which is in turn a major predictor of treatment outcomes [28]. These EFV dose reduction recommendations are therefore important for sustained efficacy of EFV in HIV management but may also lead to improved quality of life among HIV patients receiving EFV based ART.

In summary based on the current population pharmacokinetic analysis and simulation study, we propose CYP2B6 genotype based EFV dosage adjustment for HIV/AIDS patients in Uganda and the entire sub-Saharan Africa. While our recommended daily doses of 450 mg and 300 mg might meet the EFV treatment needs of HIV-1 infected Ugandans in general and individuals homozygous for variant CYP2B6*6 allele respectively, there is need for caution in events where known drug-drug interactions suspected. We recommend a large multinational clinical trial across the sub-Saharan Africa to validate the EFV dose recommendations by the current study.
